# Addressing Uganda's mental health system gaps: an urgent call for reform

**DOI:** 10.11604/pamj.2025.52.75.49455

**Published:** 2025-10-17

**Authors:** Eric Nzirakaindi Ikoona, Lucy Namulemo, Ronald Kaluya, Rebecca Ikoona

**Affiliations:** 1 National Public Health Agency, Freetown, Sierra Leone,; 2 Foothills Community-Based Interventions, Monticello, Kentucky, USA,; 3 Lindsey Wilson College, School of Professional Counseling, Kentucky, USA,; 4 Uganda Counseling and Support Services, Kampala, Uganda,; 5 Makerere University, Johns Hopkins Research Collaboration, Kampala, Uganda

**Keywords:** Uganda, mental health policy, suicide prevention, health system reform, task-shifting, digital psychotherapy

## Abstract

Uganda's mental health services are at a breaking point. Suicide ideation affects 10.6% of the population, nearly triple the global average, while Butabika National Referral Hospital, the country's only national psychiatric facility, is overcrowded, under-resourced, and unsafe. The national helpline is dominated by crisis calls about suicidal distress, and reports of abuse, forced sedation, and neglect highlight governance failures. Vulnerable groups, including HIV-positive adolescents, older adults, and women facing violence, are disproportionately underserved. Yet Uganda spends less than 1% of its health budget on mental health. Evidence from Ugandan-led innovations shows reform is both feasible and cost-effective: mobile psychotherapy reduced youth depression with over 85% engagement, and task-shifting increased depression detection by 37%. Globally, every dollar invested in treatment for depression and anxiety yields four in productivity gains, while untreated depression costs Uganda nearly US$390 million annually. Criminalisation of attempted suicide further undermines care, deterring those most in need. The path forward is clear: increase funding, decriminalize suicide, scale digital and community-based models, and strengthen data systems. Uganda does not lack evidence or solutions; it lacks political will. Without urgent reform, the human and economic toll will escalate further.

## Commentary

Uganda's mental health system is in crisis. Butabika National Referral Hospital, the country's only national psychiatric facility, is emblematic of the strain: its helpline is overwhelmed by calls related to suicidal distress [1], while overcrowding, staff shortages, and unsafe practices such as forced sedation and arbitrary restraint have been documented [2]. Far from serving as a place of healing, Butabika too often mirrors the failures of a system that invests less than 1% of the national health budget into mental health [3].

The Uganda Genome Resource revealed the scale of the challenge: 10.6% of participants reported suicidal ideation, nearly three times the global average [4]. The crisis is not evenly distributed. HIV-positive adolescents are significantly more likely to attempt suicide compared to HIV-negative peers [5]. Older adults are misdiagnosed due to ageist assumptions, leaving depression untreated [6]. Women experiencing intimate partner violence bear psychological burdens rarely recognised in clinical settings. The combination of underfunding, misdiagnosis, and stigma entrenches inequities and sustains preventable suffering. Yet, there are solutions. Ugandan researchers have tested mobile psychotherapy for young people, showing significant reductions in depression with high engagement [7]. Task-shifting models, training over 500 community health workers to use the PHQ-9 screening tool, raised detection and referral rates of depression by 37% [8]. These pilots demonstrate that reforms are not about discovering new models; they are about scaling tested, effective interventions.

The economic argument strengthens the case for reform. WHO estimates that mental disorders account for 13% of disability-adjusted life years in sub-Saharan Africa [9]. In Uganda, untreated depression drains an estimated US$390 million annually from the economy. By contrast, every US$1 invested in treatment for depression and anxiety returns US$4 in improved productivity and health [10]. Neglecting mental health is not only a moral failure, but it is also economic malpractice.

Uganda's Penal Code worsens the crisis by criminalising attempted suicide, deterring individuals from seeking help and pushing vulnerable people into prisons instead of clinics [4,6]. Decriminalisation is not radical; it is the bare minimum for a humane rights-based mental health system. Combined with increased financing, system-wide integration, and robust health information systems, reform can transform mental health services. The path is clear, but the cost of inaction grows each year. Uganda does not lack evidence or models. It lacks political will. Unless leaders act decisively to increase funding, reform laws, and scale proven interventions, lives will continue to be lost, and economic losses will mount. Every year of delay means more preventable deaths, wasted potential, and greater costs. Uganda must act now.
